# Identification of Antimicrobial Compounds in Two *Streptomyces* sp. Strains Isolated From Beehives

**DOI:** 10.3389/fmicb.2022.742168

**Published:** 2022-02-03

**Authors:** Fernando Santos-Beneit, Ana Ceniceros, Athanasios Nikolaou, José A. Salas, Jorge Gutierrez-Merino

**Affiliations:** ^1^Department of Chemical Engineering and Environmental Technology, School of Industrial Engineering, University of Valladolid, Valladolid, Spain; ^2^Departamento de Biología Funcional, Universidad de Oviedo, Oviedo, Spain; ^3^Instituto Universitario de Oncología del Principado de Asturias (I.U.O.P.A), Universidad de Oviedo, Oviedo, Spain; ^4^Instituto de Investigación Sanitaria del Principado de Asturias (ISPA), Oviedo, Spain; ^5^School of Biosciences and Medicine, University of Surrey, Guildford, United Kingdom

**Keywords:** *Streptomyces*, honey, pollen, beehive, undecylprodigiosin, antimycin, candicidin, antifungal

## Abstract

The World Health Organization warns that the alarming increase in antibiotic resistant bacteria will lead to 2.7 million deaths annually due to the lack of effective antibiotic therapies. Clearly, there is an urgent need for short-term alternatives that help to alleviate these alarming figures. In this respect, the scientific community is exploring neglected ecological niches from which the prototypical antibiotic-producing bacteria Streptomycetes are expected to be present. Recent studies have reported that honeybees and their products carry *Streptomyces* species that possess strong antibacterial activity. In this study, we have investigated the antibiotic profile of two Streptomycetes strains that were isolated from beehives. One of the isolates is the strain *Streptomyces albus* AN1, which derives from pollen, and shows potent antimicrobial activity against *Candida albicans*. The other isolate is the strain *Streptomyces griseoaurantiacus* AD2, which was isolated from honey, and displays a broad range of antimicrobial activity against different Gram-positive bacteria, including pathogens such as *Staphylococcus aureus* and *Enterococus faecalis*. Cultures of *S. griseoaurantiacus* AD2 have the capacity to produce the antibacterial compounds undecylprodigiosin and manumycin, while those of *S. albus* AN1 accumulate antifungal compounds such as candicidins and antimycins. Furthermore, genome and dereplication analyses suggest that the number of putative bioactive metabolites produced by AD2 and AN1 is considerably high, including compounds with anti-microbial and anti-cancer properties. Our results postulate that beehives are a promising source for the discovery of novel bioactive compounds that might be of interest to the agri-food sector and healthcare pharmaceuticals.

## Introduction

Many of the antibiotics currently in use are naturally derived from actinomycetes (Janardhan et al., 2014), a group of Gram-positive, spore forming, filamentous bacteria commonly found in soil. For many decades, actinomycetes have been isolated and screened from soil samples, especially strains of the genus *Streptomyces*, a very prolific antibiotic producer (Watve et al., 2001). The first antibiotics to be discovered from *Streptomyces* were actinomycin and streptothricin in the early 1940s (Waksman and Woodruff, 1941, 1942), and after that year and until the 1970s, we experienced an unprecedented exponential increase in the number of novel antimicrobials produced by *Streptomyces*, including streptomycin, neomycin or tetracycline, among others (Watve et al., 2001). Since then, the discovery of new antibiotics has dramatically decreased, and even more concerning, it is the concomitant increase of antibiotic-resistant bacteria (Mojica et al., 2021). Therefore, scientists are starting to search for Streptomycetes in other areas rather than soil, including insects and pollinators such as honeybees (Khan et al., 2020). Honeybees live in very dense colonies in beehives, where the microbial diversity is affected by their foraging activities as well as other external factors such as the habitat (local plants, water, soil, wildlife, etc.) and environment (weather, air, pollutants, etc.) (Anderson et al., 2011). Only very recently, diverse *Streptomyces* strains have been isolated from different bee species (Promnuan et al., 2021) and various parts of beehives, in particular from pollen stores (Kim et al., 2019; Grubbs et al., 2021). Most of these strains have the ability to inhibit pathogens of plants and bees (Kim et al., 2019; Grubbs et al., 2021), which could be beneficial for the health of these organisms. Here, we have used liquid chromatography and mass spectrometry to analyze the antibiotic profile of two Streptomycetes that were isolated from beehives in South East England. These isolates include *Streptomyces albus* AN1 and *Streptomyces griseoaurantiacus* AD2, two strains that were present in pollen and honey, respectively. Our study shows that both strains produce antibiotics that are effective against bacteria and yeasts, and that beehives are a promising source for the identification of novel antibiotics.

## Materials and Methods

### Growth Conditions and Isolation of Streptomycetes

We isolated the two *Streptomyces* strains from samples of honey and pollen that were collected from beehives located in South East England (Santorelli et al., 2021). Briefly, beekeepers helped us remove honeycomb frames from which 10 g of honey or pollen were directly collected using sterile swab tubes and/or containers. The collected fresh samples were immediately tightly sealed into plastic bags to prevent contamination and transported to our facilities at Surrey University, where 100 mg of the samples were diluted using 900 μL of a maximum recovery diluent (MRD) solution (Oxoid formula of peptone 1 g L^–1^; sodium chloride 8.5 g L^–1^; pH 7) with glycerol at 20% and stored in a −80°C freezer. Sample aliquots of 100 μL were then spread onto M3 agar plates, a selective and low nutrient media for the isolation of Streptomycetes (Zhang et al., 2008), and incubated at 30°C for 1–2 weeks. Colonies were visually identified as actinomycetes (or Streptomycetes) based on their morphology and transferred to Tryptic Soy Broth (TSB) with Nalidixic Acid at 25 μg mL^–1^ to minimize bacterial contamination (Santos-Beneit et al., 2014). TSB cultures were propagated at 30°C in an orbital shaker at 250 rpm for 3–4 days and the resulting mycelia spread on Mannitol Soya (MS) agar plates. MS agar plates were finally placed in a 30°C incubator for 7 days to generate spores, which were collected using a 20% glycerol MRD solution and stored at –80°C (Santos-Beneit, 2018). For growth of the *Streptomyces* isolates in liquid media the following broths were used: R5A (Fernández et al., 1998), Bennet (Mejia et al., 1998), and SM17 (EntreChem SL, personal communication).

### DNA Extraction and 16S rRNA Sequencing

DNA extraction was carried out from TSB cultures using the EZNA bacterial DNA kit according to the manufacturer’s instructions (Omega Bio-Tek, Doraville, CA, United States). Samples were left for 30 min instead of the recommended 10 min for the first incubation with lysozyme at 37°C. The products were tested for high DNA quality using a spectrophotometer and sent to LGC Genomics GmbH for 16S rRNA sequencing. The 16S rRNA gene amplicon sequencing was performed on an Illumina MiSeq sequencer using universal 16S rRNA bacterial primers for V3–V4 regions. The 16S rRNA analysis determined that two of our isolates (AD2 and AN1) were *Streptomyces*, therefore, their corresponding species were identified using BLASTN with a match greater or equal to 98% sequence identity and 100% coverage of the query (Zhang et al., 2000).

### Genome Sequencing and Analysis

DNA sequencing was performed by MicrobesNG (University of Birmingham, United Kingdom) using the Illumina MiSeq platform as previously described (Bravo et al., 2019; Stedman et al., 2020). Briefly, the library was prepared with the 250 Nextera*™* XT Library Prep Kit Genome sequencing and the quality of the generated reads was trimmed with Trimmomatic (Bolger et al., 2014). The generated contigs were assembled from the paired-end reads using Shovill version 1.0.4^1^ with SPAdes 3.13.0 (Bankevich et al., 2012) and the resulting genome assemblies were verified by N50 and L50 using Quast version 4.5 (Gurevich et al., 2013) and annotated with Prokka version 1.13 (Seemann, 2014). Further characterization was then carried out using several bioinformatics tools such as BLAST, Phylophlan (Segata et al., 2013) and StrainSeeker (Roosaare et al., 2017). The latter two allow for fast species identification through genome comparison with closely related bacterial strains. Sequencing reads, genome assemblies and metadata have been uploaded onto Genbank in BioProject PRJNA746445. Subsequent computer-aided database searching and comparative genome analyses were carried out with reference strains such as *S. albus* J1074 (Chater and Wilde, 1976) using the bioinformatic tool antiSMASH 6.0 (Medema et al., 2011; Blin et al., 2013, 2021) and the BLAST program (Altschul et al., 1997).

### Growth Inhibition Bioassays

**FIGURE 1:**
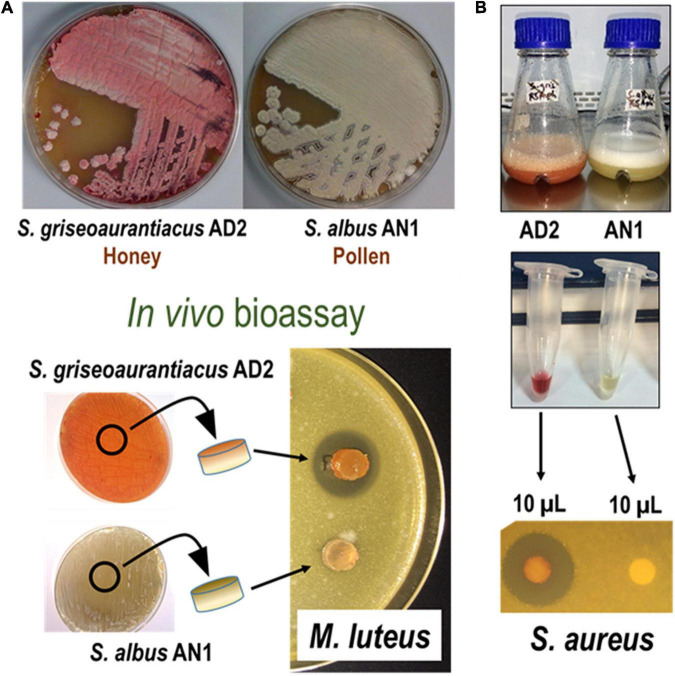
Phenotypic and antibiotic activity characterization of *Streptomyces albus* AN1 and *Streptomyces griseoaurantiacus* AD2. The figure shows two bioassays in which live cells **(A)** and cell extracts **(B)** derived from both strains are tested against *Micrococcus luteus* or *Staphylococcus aureus*. Same results were observed using *Bacillus cereus*, *Enterococus faecalis*, and *Streptomyces coelicolor*.

The two isolates were assessed for growth inhibition against different types of indicator microorganisms, including *Micrococcus luteus* ATCC 23262, *Bacillus cereus* ATCC 14579, *Enterococus faecalis* ATCC 33186, *Staphylococcus aureus* BAA-1747, *Streptomyces coelicolor* M145, *Escherichia coli* ESS, *Pseudomonas aeruginosa* ATCC 10145, *Salmonella enterica* subsp. *enterica* serovar Typhimurium ATCC14028, *Candida albicans* ATCC 32077, and *Saccharomyces cerevisiae* VL6-48. The bioactivity assays were performed *in vivo* (*Streptomyces* vs. indicator) and *in vitro* (organic extract from *Streptomyces* vs. indicator). For the *in vivo* assays we directly placed a small portion of an agar containing *Streptomyces* cells on top of agar plates (LB or TSA) inoculated with a lawn of the indicator microorganism (Figure 1A). The *in vitro* assays were conducted using cell extracts from cultures of the *Streptomyces* strains in different broths (see next section). These cell extracts were soaked in sterile disks that were deposited on agar plates seeded with the indicator microorganism (Figure 1B). Activity was examined as visible inhibition halos after incubation of the agar plates at 30°C for several days, depending on the indicator organism used in the assay. Inhibition was generally observed after 1 or 2 days of incubation and was stable for several weeks.

### Analysis of Metabolites by Ultra Performance Liquid Chromatography and Liquid Chromatography–Mass Spectrometry

Metabolites from cultures of the isolated strains were extracted using two different organic solvents (ethyl acetate or 1-butanol). The cultures were diluted with the organic solvents at a ratio of 1:1 and maintained in constant mixing at room temperature for 2 h. The organic phase was then separated from the aqueous phase by centrifugation and collected for further evaporation using a SpeedVac. The resulting extracts were reconstituted in methanol and analyzed by reversed phase chromatography using acetonitrile and water containing 0.1% trifluoroacetic acid (TFA) as solvents in an Acquity UPLC instrument fitted with a BEH C18 column (1.7 μm, 2.1 × 100 mm; Waters). Samples were eluted with 10% acetonitrile for 1 min, followed by a linear gradient from 10 to 100% acetonitrile over 7 min, at a flow rate of 0.5 mL min^–1^ and a column temperature of 35°C. For LC-MS analysis, we first used an Alliance chromatographic system coupled to a ZQ4000 mass spectrometer and a SunFire C18 column (3.5 μm, 2.1 × 150 mm; Waters). Solvents were the same as above and elution was performed with an initial isocratic hold with 10% acetonitrile for 4 min followed by a linear gradient from 10 to 88% acetonitrile over 26 min, at 0.25 mL min^–1^. MS was then carried out by electrospray ionization in the positive mode, with a capillary voltage of 3 kV and a cone voltage of 20 and 50 V. Detection and spectral characterization of the peaks obtained from UPLC and LC-MS were performed by photodiode array detection using an empower software (Waters) to extract bidimensional chromatograms at different wavelengths, normally within the range between 200 and 500 nm depending on the spectral characteristics of the desired compound.

### Dereplication Studies (LC-DAD-HRMS)

Some organic extracts were subjected to dereplication using a combination of LC-DAD-HRMS analysis (Perez-Victoria et al., 2016) with an Agilent 1200 Rapid Resolution HPLC system coupled to an ESI mode Bruker maXis mass spectrometer. The selected samples included extracts obtained with 1-butanol from cultures of *S. griseoaurantiacus* AD2 and *S. albus* AN1 in R5A cultures as well as those extracted with ethyl acetate from cultures of *S. albus* AN1 in Bennet. Separation of compounds was then conducted using acetonitrile and water containing ammonium formate 13 mM and 0.01% TFA as solvents in a Zorbax SB-C8 column (3.5 μm, 2.1 × 30 mm). The resulting chromatographic runs were processed using a Bruker’s in-house component extraction algorithm to identify Total Ion Chromatogram (TIC) positive peaks at 210 nm. For molecular formula interpretation, we used retention time and exact mass as search criteria on the high-resolution mass spectrometry database of Medina Foundation (Granada, Spain). If a possible match was found, the molecule was labeled as a suggested compound, together with the producing organism and the wavelength spectrum. For those compounds with no matches in the above database, the search for the exact mass/molecular formula was performed using the Chapman and Hall’s Dictionary of Natural Products (DNP).

## Results

Samples collected from honey and pollen stores of beehives located in South East England were screened for the presence of Streptomycetes. Enrichment isolation for *Streptomyces* species resulted in the identification of three isolates (AD1, AD2, and AN1) from all sampled material (Santorelli et al., 2021, under review). AD1, isolated from honey, is also included under bioproject PRJNA746445. This isolate did not show any relevant antimicrobial activity using the experimental conditions described in this study and was not further studied (see Supplementary Table 1 and Supplementary Figure 1). 16S rRNA sequencing analysis informed that the three isolates were *Streptomyces*, with a match equal to 99% sequence identity and 100% coverage of the query. At species level, isolates AD1 and AD2 were clearly identified as *Streptomyces drozdowiczii* and *Streptomyces griseoaurantiacus*, respectively, and this was corroborated by genome BLAST. However, results for AN1 were inconclusive (Supplementary Table 1). This isolate was initially identified as *Streptomyces* sp. strain 632F, with a 100% sequence identity to *S. sampsonii*, but also 99% identical to *S. albidoflavus, S. albus*, *S. champavatii, S. fungicidicus, S. griseus*, and *S. somaliensis*. AN1 was finally confirmed to be *S. albidoflavus* using BLAST, Phylophlan and StrainSeeker, with a high phylogenetic similarity to *S. albus* J1074, which has recently been reclassified as *S. albidoflavus* (Labeda et al., 2014). Nevertheless, to facilitate comparison with strain J1074, AN1 has been temporarily referred to as *S. albus*. Following their identification, we proceeded to check whether our beehive isolates inhibit different microorganisms, in particular, yeasts and Gram-positive and Gram-negative bacteria.

**FIGURE 2:**
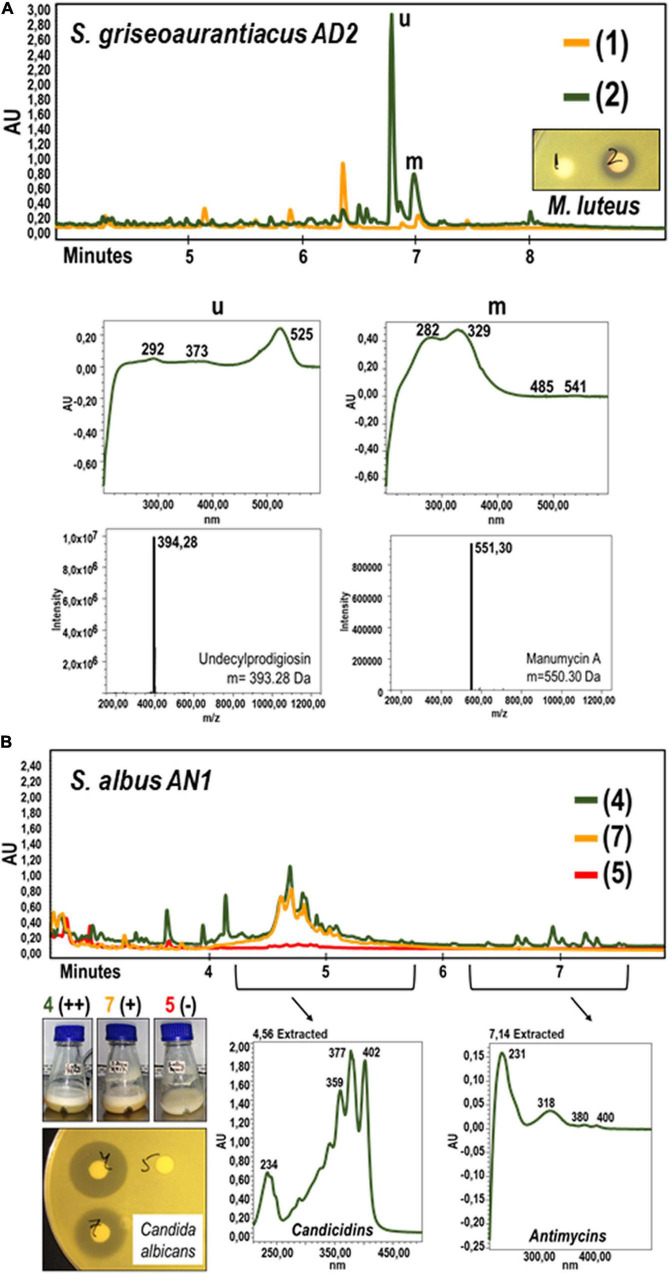
Metabolic characterization by UPLC and LC-MS analyses of cell extracts from cultures of *Streptomyces griseoaurantiacus* AD2 **(A)** and *Streptomyces albus* AN1 **(B)** strains. **(A)** *S. griseoaurantiacus* AD2 extracts were generated from 96 h-cultures in R5A (green) or SM17 (orange), and assayed against *M. luteus*. The graphs below the UPLC chromatograms show the UV spectrum of the metabolites that might display antimicrobial activity (see peaks named as “u” and “m”). The corresponding molecular weights were calculated by LC-MS analyses (and also by dereplication analyses), resulting in the identification of the metabolites undecylprodigiosin and manumycin A. **(B)** *S. albus* AN1 extracts were generated from 96 h-cultures in R5A (green), SM17 (orange) and Bennet (red), and assayed against *C. albicans*. The peaks selected from the AN1 cultures possess an UV spectrum that match that of the candicidins and antimycins.

**FIGURE 3:**
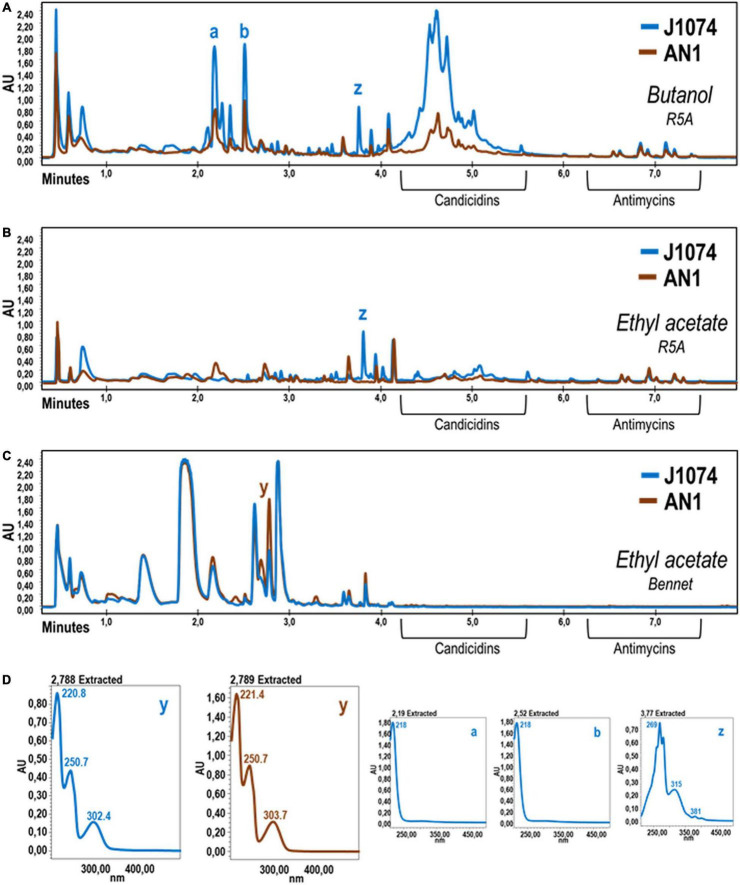
Comparative UPLC chromatograms of cell extracts obtained with several solvents using cultures of *S. albus* AN1 (brown) and J1074 (blue) in different broths. The two strains were grown in R5A **(A,B)** or Bennet **(C)** broth for 96 h and their corresponding metabolites extracted with either 1-butanol **(A)** or ethyl acetate **(B,C)**. Note that, when both strains are grown in R5A, the candicidins are hardly detected with the ethyl acetate extraction procedure. **(D)** UV spectra of differential peaks (a, b, y, and z) are shown. UV spectra of peaks a-b and those in between (not shown) were almost identical. These molecules were unable to be dereplicated and therefore no specific activity could be attributed to these putatively unknown molecules.

The *in vivo* bioassays showed that *S. griseoaurantiacus* AD2 is able to inhibit the growth of several Gram-positive bacteria, including *Micrococcus luteus*, *Bacillus cereus*, *Enterococus faecalis*, *Staphylococcus aureus*, and *Streptomyces coelicolor* (Figure 1A). However, it was unable to inhibit the growth of either Gram-negative bacteria (*Escherichia coli*, *Pseudomonas* sp., or *Salmonella* sp.) or yeasts (*Candida albicans* or *Saccharomyces cerevisiae*). On the other hand, *S. albus* AN1 displayed antifungal activity but lacked any inhibitory effect against both Gram-positive and Gram-negative bacteria (Figure 1A and Supplementary Figure 1A). AN1 was capable of inhibiting Gram-positive bacteria only under certain growth conditions, for example using Bennet medium (Supplementary Figure 1B). Identical results were observed with the laboratory reference strain *S. albus* J1074 (Supplementary Figure 1). Additionally, the *in vitro* bioassays showed that the antimicrobial activity displayed by AD2 and AN1 depended on the broth media and extraction method (ethyl acetate or 1-butanol). As illustrated in Figures 2, 3 and Supplementary Figure 1, the best antibiotic profile was observed with 1-butanol and R5A, a broth that has been routinely used for the activation of distinct secondary metabolites, including the antifungal compounds candicidins and antimycins produced by *S. albus* J1074 (Olano et al., 2014). The metabolites present in the extracts were then analyzed by UPLC, LC-MS, and dereplication (LC-DAD-HRMS).

The UPLC analysis on the extracts obtained from the R5A cultures of *S. griseoaurantiacus* AD2 allowed us to identify two peaks with potential antimicrobial activity against *M. luteus* (Figure 2A). These two peaks (named as “u” and “m” in Figure 2A) were absent in extracts derived from AD2 cultures in SM17, a broth where no antibacterial activity was recorded. LC-MS analysis resulted in the identification of two metabolites with a mass of *m/z* 394.28 [M + H]^+^ and *m/z* 551.30 [M + H]^+^ (Figure 2A), which were further confirmed to be undecylprodigiosin (C_25_H_35_N_3_O) and manumycin A (C_31_H_38_N_2_O_7_) by dereplication. Undecylprodigiosin is a red antibiotic that confers a characteristic reddish coloration to the producer (Feitelson et al., 1985), the best-known example being the model streptomycete *S. coelicolor*. Manumycin A is known to exhibit antimicrobial activity against Gram-positive bacteria such as *S. aureus*, *E. faecalis*, and *Bacillus subtilis* (Kohno et al., 1996). Both metabolites are also produced by other *S. griseoaurantiacus* strains (Li et al., 2011; Chanadech et al., 2021). Additionally, traces of manumycin-related compounds were also detected with the dereplication studies, including manumycin D (C_31_H_40_N_2_O_7_), chinikomycin A (C_31_H_39_ClN_2_O_7_), and antibiotic TMC-1A (C_28_H_36_N_2_O_7_) (Supplementary Figure 2). Manumycin-type metabolites represent a group of several tens of structurally related small linear polyketides, which typically contain two short polyketide chains connected via a central *m*C_7_N cyclic unit derived from 3-amino-4-hydroxybenzoic acid (Zeeck et al., 1987a,b). No antimicrobial activity has been reported for most of these compounds, including the previously mentioned manumycin A derivatives (Kohno et al., 1996; Li et al., 2005).

Extracts from different cultures of *S. albus* AN1 exhibited a different antifungal spectrum, ranging from strong to moderate activity if obtained from R5A or SM17, respectively. Although extracts from Bennet displayed antibacterial activity, no antifungal activity was observed from this medium (Supplementary Figure 1). The UPLC analysis revealed that the Bennet extract lacks some peaks that were detected from the R5A and SM17 extracts after 4.5 min of retention (Figure 2B). These peaks corresponded to candicidins as previously reported based on their corresponding UV spectra (Olano et al., 2014; González et al., 2016; Hoz et al., 2017). In addition, we observed other peaks at 7.14 min, but only if extracts derive from R5A cultures, that were identified as antimycins by UV monitoring (Figure 2B). As expected, all the detected peaks correlated perfectly with the antifungal profile of AN1, which changed from zero activity in Bennet to moderate or high activity in SM17 or R5A (due to the production of only candicidins or both candicidins and antimycins, respectively). Furthermore, the UPLC chromatograms that we obtained from our strain AN1 were almost identical to those from the reference strain *S. albus* J1074; with only a few differential peaks observed from each of the cultures and extraction conditions tested (Figure 3). Candicidins and antimycins were detected in 1-butanol extracts from the two *S. albus* strains (Figure 3A). Antimycins were also recovered at the same level when the extraction was conducted with ethyl acetate. However, this solvent was less effective for the extraction of candicidins (Figure 3B). In fact, as expected, 1-butanol extracts from AN1 and J1074 were more active against *C. albicans* than those obtained with ethyl acetate, as illustrated in Supplementary Figure 1C. Curiously, ethyl acetate proved to be more efficient to extract metabolites with antimicrobial activity against *M. luteus*, especially if the extracts originate from Bennet cultures and our strain AN1 (see Supplementary Figure 1B). Whether this different antibacterial activity correlates or not with the observation of a unique differential peak in the extracts of both strains is an intriguing question that remains yet to be elucidated (see “y” in Figure 3C). Unfortunately, the LC-MS and dereplication analyses corresponding to this peak were uncertain, thus we could not interpret its enigmatic UV spectrum. Similar result was observed with other peaks that were differently produced by the two *S. albus* strains (see peaks “a,” “b,” and “z” in Figure 3C). On the other hand, the dereplication studies let us detect a plethora of several bioactive compounds from the extracts of *S. albus* cultures. These compounds differed amongst the distinct broth used (i.e., R5A or Bennet) and, in some cases, were similar to those detected from other *S. albus*(-like) strains (Table 1).

**TABLE 1:** Bioactive metabolites produced by our isolate *S. albus* AN1 by comparison with reference laboratory strain *S. albus* J1074 and other *S. albus*(-like) strains.

Metabolites^a^	Bioactivity	*S. albus* AN1^b^	*S. albus* J1074^c^	RS68, RS77, RS78^d^
Nocardamine	Antitumor/Antibacterial	Yes (BEN)	Yes	Yes
Deferoxamine	Antitumor	Yes (BEN)	No	Yes
Antimycin A	Antifungal	Yes (R5A)	Yes	No
Alteramide A	Antifungal	Yes (R5A)	Yes	Yes
Surugamides A/B/C/D/E	Antitumor/Antifungal	Yes (R5A)	Yes	Yes
Germicidin G	Germicidin	Yes (R5A)	No	Yes
N-acetyltyramine	Antitumor/Antifungal	Yes (R5A)	Yes	No
Candicidins	Antifungal	Yes* (R5A)	Yes	No
Paulomycins	Antibacterial	No	Yes	No
Salinomycin^e^	Antibacterial	No	No	No
Tirandamycin A	Antiamoebic	No	No	Yes
Fredericamycin A	Antibacterial/Antifungal	No**	No	No

*^a^Metabolites were identified by dereplication using extracts derived from different broths such as Bennet and R5A. Compounds for which the biosynthetic genes have been chemically and genetically characterized in the reference laboratory strain S. albus J1074 are highlighted with gray-shadowed boxes. Many derivatives of Antimycin A were identified in S. albus AN1 (i.e., Antimycin A3a/A3b/A7a/A7b, A11/A17/A2/A2a/A2b/A8a/A8b, A12/A13/A19/A1a/A1b).*

*^b^This study.*

*^c^In our laboratory, production of secondary metabolites by S. albus J1074 has been routinely monitored using R5A as production medium. These metabolites have been detected previously in Olano et al. (2014), González et al. (2016), and Hoz et al. (2017).*

*^d^Santamaría et al. (2020). The three strains (RS68, RS77, and RS78) were isolated from the larvae of the xylophagous coleopteran Cerambyx welensii and, similarly to our AN1 strain, were identified initially as Streptomyces sampsonii by 16S rDNA analyses.*

*^e^The cluster responsible for salinomycin production has been characterized from S. albus DSM 41398 (Yurkovich et al., 2012). Salinomycin and its derivatives exhibit high antimicrobial activity against Gram-positive bacteria, including methicillin-resistant Staphylococcus aureus, methicillin-resistant Staphylococcus epidermidis, and Mycobacterium tuberculosis. Salinomycin is inactive against fungi such as Candida albicans and Gram-negative bacteria (Antoszczak et al., 2014).*

*UV spectra of these peaks corresponded to that of the candicidins but with no coincidence on the DNP (Dictionary of Natural Products).

**The compound was predicted by antiSMASH in AN1 but not in J1074.

## Discussion

In this study, we have identified and characterized two *Streptomyces* species that derive from beehives. The consensus is that only highly resistant spore forming bacteria, such as *Clostridium* and *Bacillus* can survive the high osmolarity and acidic environment of honeybee products (Olaitan et al., 2007). However, the presence of viable Streptomycetes is also possible under these adverse conditions, as we have proved with the isolation of *S. griseoaurantiacus* from honey, and *S. albus* from pollen. Many species of *Streptomyces* are able to produce spores and resist acidic, low moisture soils (Lee and Hwang, 2002). In this respect, it should be highlighted that, for the first time, *S. griseoaurantiacus* is isolated from honey. Only a few studies had already reported the isolation of this species, but from soil, marine sediment, and agricultural waste residues (Matsumoto et al., 1998; Li et al., 2011; Kumar, 2015). We have observed that *S. griseoaurantiacus* AD2 inhibits the growth of different Gram-positive bacteria, and that this antibacterial feature could be due to its capacity to produce the antibiotics undecylprodigiosin and manumycin A. At least three different types of antibiotics (undecylprodigiosin, manumycin A, and diperamycin) have been shown to be produced by distinct *S. griseoaurantiacus* strains (Matsumoto et al., 1998; Li et al., 2011). Diperamycin, a member of cyclic hexadepsipeptide antibiotics, shows potent inhibitory activity against methicillin-resistant *Staphylococcus aureus* (MRSA); a major killer in hospitals (Matsumoto et al., 1998). This compound was not detected in cultures of our AD2 strain using the methods described in this study. On the other hand, we have found different manumycin A-derived compounds, including manumycin D, chinikomycin A and antibiotic TMC-1A, and several distinct compounds (Supplementary Table 2 and Supplementary Figure 2). Manumycin-type metabolites were first reported as antibiotics (Ali et al., 1999), but later they have mostly been studied for their antitumor activity, with over 350 studies published on the topic so far (Gorniaková et al., 2021). These compounds specifically inhibit Ras-farnesyl transferase and induce the production of reactive oxygen species (ROS), leading to cell proliferation inhibition and apoptosis (Hara et al., 1993; She et al., 2006). Promising anticancer compounds have also been identified from the *S. albus* AN1 isolate, including nocardamine, deferoxamine, and surugamides (Table 1), as previously reported with the reference strain J1074 (Song et al., 1994; Zhao et al., 2006, 2010; Liu et al., 2009; Olano et al., 2014; González et al., 2016; Hoz et al., 2017). In particular, surugamides have been shown to exhibit not only good antitumor activity but also significant antifungal activity against some yeast such as *S. cerevisiae* (Xu et al., 2017). Additionally, we have also detected antifungal metabolites from cultures of AN1, including candicidins, antimycins, alteramides, and N-acetyltyramine. Our dereplication analyses suggest that the number of putative bioactive metabolites produced by AN1 could be even higher (Supplementary Table 2), with some of them being novel since no coincidences on the DNP (Dictionary of Natural Products) have been obtained. No fractionating of the extracts was performed in the AN1 strain but, for most of these compounds, it was previously performed in the reference strain J1074 under the same laboratory conditions of this study (Olano et al., 2014; González et al., 2016; Hoz et al., 2017). Actually, the metabolic wealth of our isolated AN1 strain is almost identical to that of the reference strain *S. albus* J1074 (Table 1).

Interestingly, the antifungal activity displayed by both *S. albus* AN1 and J1074 strains varied depending on the culture broth and the organic solvent used for the metabolic extraction. We have seen that candicidins were hardly extracted with ethyl acetate, whereas antimycins were recovered with either ethyl acetate or 1-butanol. On the other hand, we have noticed a strong nutritional regulation on the production of both candicidins and antimycins. In fact, we only found antimycins in one of the three broth media tested. This observation is not unexpected since it has been known for a long time that culture media, in particular the carbon, nitrogen and phosphate sources, influence significantly microbial growth and metabolism (Martín, 2004; Santos-Beneit, 2015; Pan et al., 2019). Furthermore, we have also recorded some interesting differences between the metabolic profiles of AN1 and J1074 strains. According to our dereplication studies, AN1 produces deferoxamine and germicidin G, two metabolites that have not been yet detected in J1074 (Table 1). Deferoxamine is a derivative of the antitumoral cyclic siderophore nocardamine and seems to be useful in the treatment of acute iron intoxication and for various ecological remediation strategies (Holden and Nair, 2019). Germicidin G is the first known autoregulative inhibitor of spore germination in *Streptomyces* (Aoki et al., 2011). Strikingly, these two compounds are produced by different *S. sampsonii* strains (RS68, RS77, and RS78; see Table 1) that are very similar to our isolated AN1 strain. These strains have recently been isolated from the beetle species *Cerambyx welensii* (Santamaría et al., 2020).

Some of the metabolic similarities described for *S. albus* AN1 and J1074 have been confirmed by genome analysis (Supplementary Figure 3). The only significant difference observed between the genomes of AN1 and J1074 is the type 2 polyketide synthetase cluster illustrated at the bottom of Supplementary Figure 3. This cluster is only present in AN1 and shares an 84% of identity with fredericamycin A (MIBiG access number BGC0000224). Fredericamycin A was originally isolated from a *Streptomyces somaliensis* strain that possesses strong antibacterial and antifungal activities (Zhang et al., 2015). Interestingly, the structural variant fredericamycin C2, which has been extracted from *S. albus* subsp. *chlorinus*, also displays strong cytotoxic activity (Rodríguez-Estévez et al., 2020). Therefore, the analogs that our AN1 strain produce could be further tested as promising anticancer drugs. However, it should be noted that, under our experimental conditions, the identified fredericamycin cluster must be cryptic as no derivatives have been detected following LC-MS and dereplication analyses. Nevertheless, according to all the results shown in this study, our *S. albus* AN1 isolate postulates as powerful biological tool to combat fungal infections in agriculture.

In summary, here we describe the association of antibiotic-producing Streptomycetes with products made by honeybees in beehives, and show that these bacteria display antifungal and antibacterial activities. Very recent publications have reported that *Streptomyces* species isolated from bees and honeybee products inhibit the growth of plant and bee pathogens (Kim et al., 2019; Grubbs et al., 2021), suggesting an important beneficial role of Streptomycetes in maintaining the health of the beehives. Our study provides more encouraging information, not only to reinforce this underexplored relationship, but also to expand the search for novel antibiotics toward complex microbial environments such as beehives.

## Data Availability Statement

The datasets presented in this study can be found in online repositories. The names of the repository/repositories and accession number(s) can be found below: https://www.ncbi.nlm.nih.gov/genbank/, BioProject PRJNA746445.

## Author Contributions

FS-B conceived the work, planned, performed the experiments, carried out data analysis, and wrote the manuscript. AC contributed to the antiSMASH and mass spectrometry analyses. AN conducted antimicrobial screening experiments and aided in bacterial isolation. JS contributed to the interpretation of the results. JG-M isolated the strains, planned, conceived the work, and edited the manuscript. All authors have read the final manuscript.

## Conflict of Interest

The authors declare that the research was conducted in the absence of any commercial or financial relationships that could be construed as a potential conflict of interest.

## Publisher’s Note

All claims expressed in this article are solely those of the authors and do not necessarily represent those of their affiliated organizations, or those of the publisher, the editors and the reviewers. Any product that may be evaluated in this article, or claim that may be made by its manufacturer, is not guaranteed or endorsed by the publisher.

## Footnotes

1
https://github.com/tseemann/shovill
