# Supplementary material for: Identification of Antimicrobial Compounds in Two Streptomyces sp. Strains Isolated From Beehives
Source: Front Microbiol. 2022 Feb 3;13:742168. doi: 10.3389/fmicb.2022.742168 (PMC8851239; doi:10.3389/fmicb.2022.742168)

Supplementary Material

Supplementary Material

1 Supplementary Tables

Supplementary Table S1 shows the 16S rRNA sequencing species identification for each of the strains isolated from beehives in this study.

**sample**	**description**	**ident**	**acc.no**
AD1	Streptomyces drozdowiczii partial 16S rRNA gene, isolate PhyCEm-1258	99%	AM921645.1
AD2	Streptomyces griseoaurantiacus strain NBRC 15440 16S ribosomal RNA gene, partial sequence	99%	NR_041186.1
AN1	Streptomyces sp. strain 632F 16S ribosomal RNA gene, partial sequence	99%	KX426371.1

Supplementary Table S2. Other metabolites (no mentioned in the text) identified by dereplication using extracts derived from *S. griseoaurantiacus* AD2 and *S. albus* AN1 cultures.

Strain	Chemical formula	Compound	UV spectrum	Structure
AD2	$C_9H_9NO_4$	UO-119	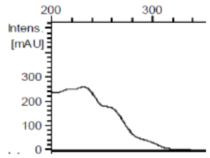	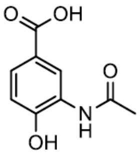
AD2	$C_9H_8N_2O$	4-Hydroxy-2-methylquinazoline	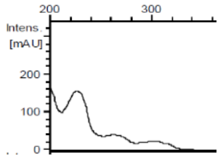	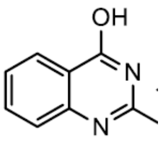
AD2	$C_{17}H_{14}N_2O_6$	Griseolutein A	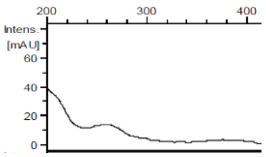	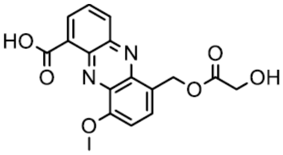

AD2	$C_{18}H_{29}NO_7S$	No coincidence on the DNP	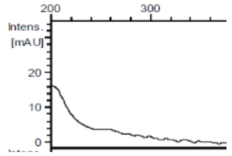	
AD2	$C_{17}H_{13}NO_7$	No coincidence on the DNP	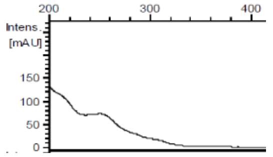	
AD2	$C_{23}H_{22}N_2O_9S$	No coincidence on the DNP	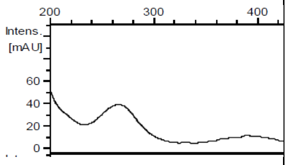	
AD2	$C_{19}H_{17}NO_7$	No coincidence on the DNP	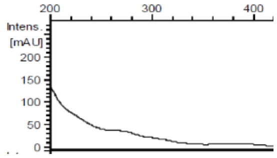	
AD2	$C_{19}H_{15}NO_7$	No coincidence on the DNP	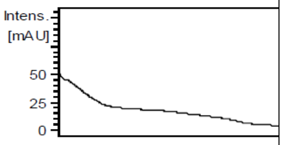	
AN1	$C_{13}H_{22}O_4$	Six coincidences on the DNP	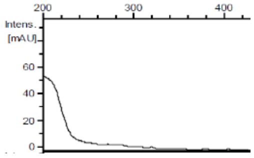	
AN1	$C_{10}H_{13}NO_3$	Three coincidences on the DNP:<br>i) Venezueline G<br>ii) N-Salicyloyl-2-amino-1-propanol<br>iii) 2-Amino-3-(4-hydroxymethylphenyl) propanoic acid	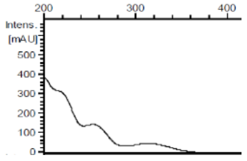	
AN1	$C_{33}H_{56}N_2O_8$	No coincidence on the DNP	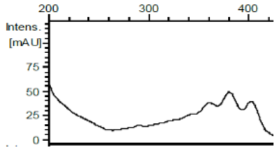	

2 Supplementary Figures

Supplementary Figure S1. Antimicrobial *in vivo* **(A-B)** and *in vitro* **(C)** bioassays of the *Streptomyces* isolated from beehives against different microorganisms (AD1 strain is also included under bioproject PRJNA746445 but not further studied). Cells were cultivated on R5A **(A)** or different agar media **(B)**, while extracts were obtained from R5A broth using either butanol or ethyl acetate (Ethyl Ac).

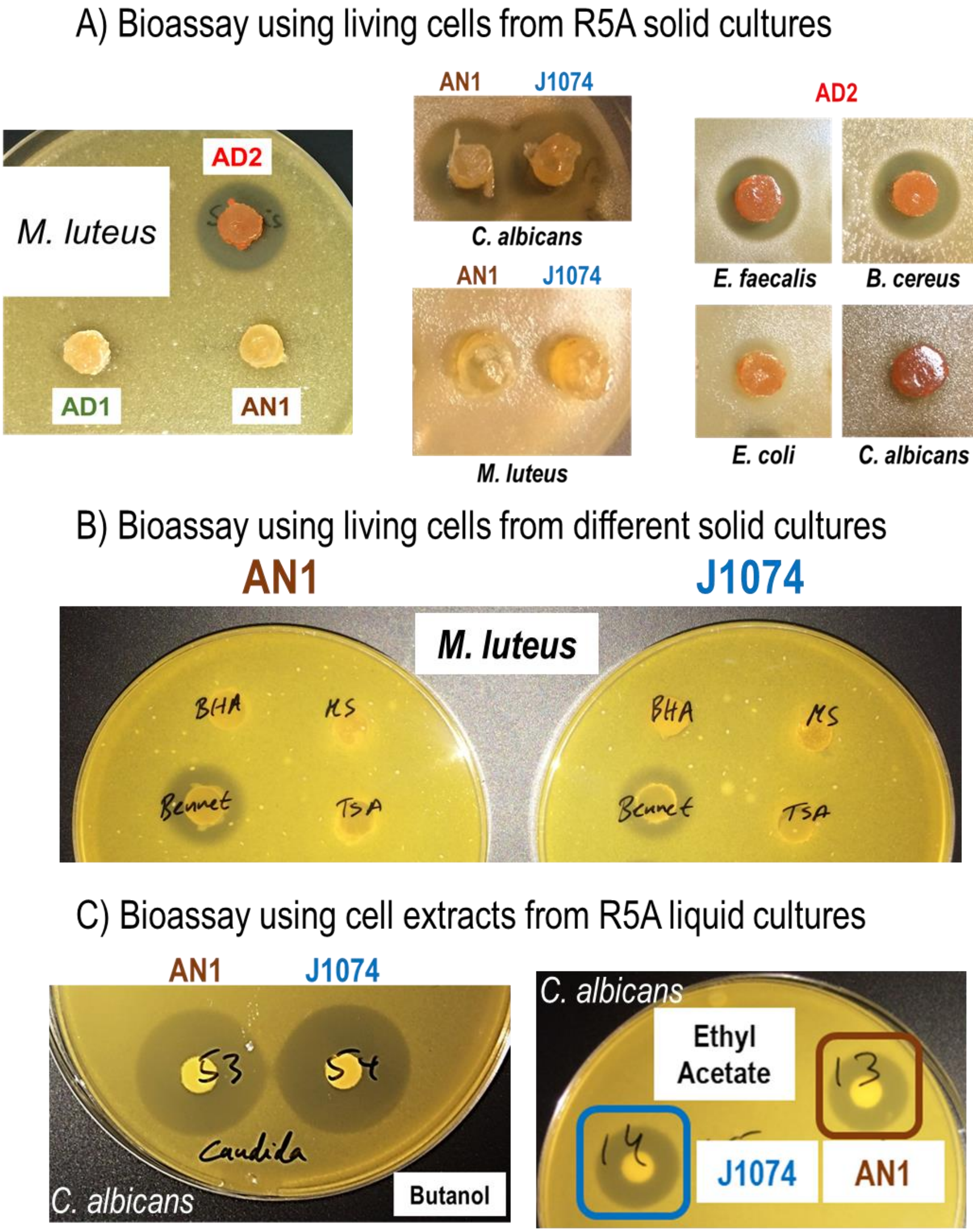

Supplementary Figure S2. Manumycin derivatives detected by replication in 1-butanol extracts from *Streptomyces griseoaurantiacus AD2* R5A cultures.

A) Manumycin D

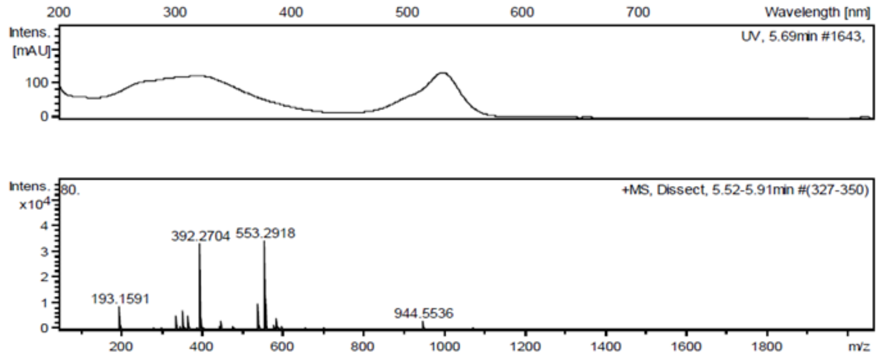

$C_{31}H_{40}N_2O_7$

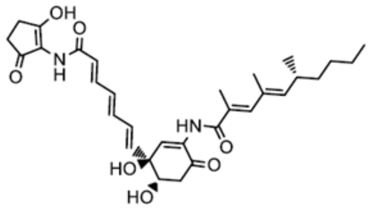

B) Chinikomycin A

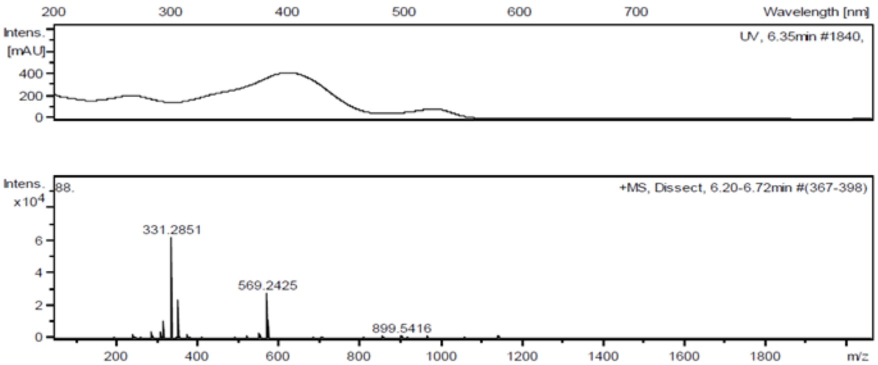

$C_{31}H_{37}ClN_2O_6$

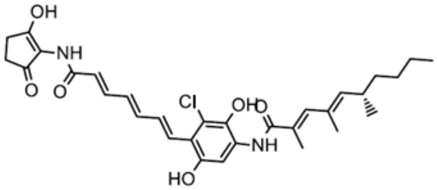

C) Antibiotic TMC 1A

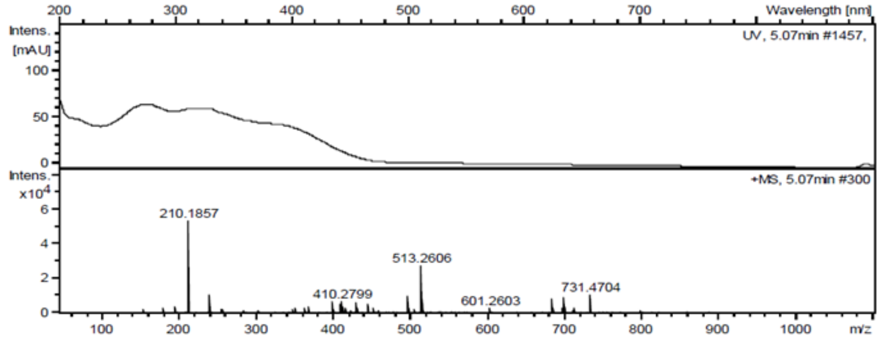

$C_{28}H_{36}N_2O_7$

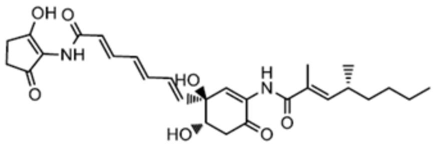

Supplementary Figure S3. Secondary metabolite biosynthetic clusters identified by antiSMASH. Note a cluster showing 84% similarity to the known biosynthetic gene cluster of fredericamycin A.

AntiSMASH overview of *Streptomyces albus* AN1

Identified secondary metabolite regions using strictness 'relaxed'

Region	Type	From	To	Most similar known cluster		Similarity
Region 12.1	NRPS	1	30,724	surugamide A / surugamide D	NRP	19%
Region 14.1	T2PKS	1	29,531	fredericamycin A	Polyketide:Type II	84%
Region 16.1	NRPS	1	27,884	dechlorocuracomycin	NRP	20%
Region 28.1	T1PKS	1	22,458			
Region 38.1	NRPS	1	20,427			
Region 44.1	T1PKS	1	19,551			
Region 53.1	T3PKS	1	18,536	herboxidiene	Polyketide	6%
Region 54.1	RiPP-like	7,270	18,073			
Region 81.1	RRE-containing	852	15,546	surugamide A / surugamide D	NRP	38%
Region 95.1	NRPS	1	14,170			
Region 101.1	T1PKS , NRPS	1	13,648	antimycin	NRP:Cyclic depsipeptide + Polyketide:Modular type I	13%
Region 113.1	ectoine	1,765	12,163	ectoine	Other	100%
Region 129.1	terpene	320	12,234	hopene	Terpene	46%
Region 152.1	siderophore	1	7,011			
Region 173.1	terpene	1	10,395	isorenieratene	Terpene	25%
Region 180.1	terpene	1	10,213	albaflavenone	Terpene	100%
Region 295.1	T1PKS	1	7,455	candicidin	Polyketide	28%
Region 311.1	NRPS	1	7,041	antimycin	NRP:Cyclic depsipeptide + Polyketide:Modular type I	13%
Region 343.1	lanthipeptide-class-iii	1	6,537			
Region 350.1	lanthipeptide-class-ii	1	6,368			
Region 364.1	siderophore	1	6,192			
Region 420.1	NRPS-like	1	5,409	heat-stable antifungal factor	NRP + Polyketide	37%
Region 475.1	NRPS-like	1	4,829			
Region 512.1	lanthipeptide-class-iii	1	4,488	AmfS	RiPP:Lanthipeptide	40%
Region 552.1	RRE-containing	1	4,133			
Region 717.1	terpene	1	2,813	hopene	Terpene	15%
Region 786.1	terpene	1	2,351	geosmin	Terpene	100%
Region 871.1	RiPP-like	1	1,874			
Region 953.1	NRPS-like	1	1,554	rhizomide A / rhizomide B / rhizomide C	NRP	100%
Region 1012.1	terpene	1	1,375			

Cluster for fredericamycin A predicted by antiSMASH in *S. albus* AN1 but not in *S. albus* J1074

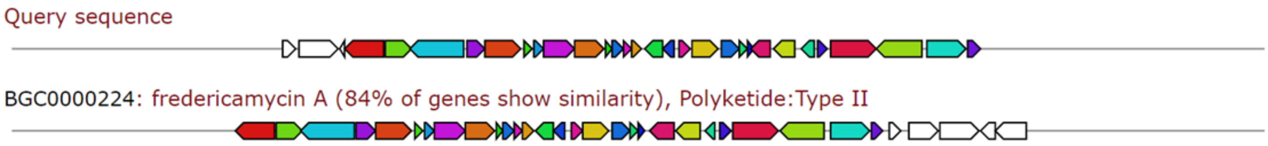

Supplement: Supplementary file 1 [file Data_Sheet_1.PDF]
